# Effect of single spinal anesthesia with two doses ropivacaine on urinary retention after hemorrhoidectomy in male patients

**DOI:** 10.3389/fsurg.2022.1077575

**Published:** 2023-01-11

**Authors:** Lei-lei Wang, Meng Kang, Li-xin Duan, Xu-fei Chang, Xiao-xin Li, Xiang-yang Guo, Zhi-yu Kang, Yong-zheng Han

**Affiliations:** ^1^ Department of Anesthesiology, Peking University Third Hospital Yanqing Hospital, Beijing, China; ^2^ Department of Anesthesiology, Peking University Third Hospital, Beijing, China; ^3^ Department of General Surgery, Peking University Third Hospital Yanqing Hospital, Beijing, China

**Keywords:** ropivacaine, single spinal anesthesia, hemorrhoidectomy, postoperative uroschesis, male patients

## Abstract

**Background:**

Anorectal diseases are common in the population and include internal, external, and mixed hemorrhoids. Although hemorrhoid surgery is a brief operation, anesthesia, anesthetic drugs, drug concentrations, and anesthesia level control are closely related to postoperative uroschesis. For hemorrhoid surgery, a single spinal block with ropivacaine is commonly used that blocks the S2-S4 parasympathetic nervous system, which in turn governs the voiding reflex, causing postoperative urinary retention; this affects the recovery of patients. This study was performed to investigate the effects of two doses ropivacaine that provided satisfactory analgesia and muscle relaxation and inhibited adverse reflexes on urinary retention after hemorrhoidectomy.

**Methods:**

The study included 200 male patients who underwent anorectal surgery with American Society of Anesthesiologists (ASA) grade I–II single elective spinal anesthesia between March 2021 and March 2022. Patients were randomly assigned to 2 groups using a random number table: Group A (*n* = 100) received 10 mg 0.5% ropivacaine (1.5 ml 1% ropivacaine + 1.5 ml 10% glucose = 3 ml), and Group B (*n* = 100) received 15 mg 0.5% ropivacaine (1.5 ml 1% ropivacaine + 1.5 ml 10% glucose = 3 ml).

**Results:**

The anal sphincter exhibited good relaxation, and no obvious traction pain or significant difference in the time of muscle strength recovery was observed between the 10 mg and 15 mg 0.5% ropivacaine groups (*P *> 0.05). The 10 mg 0.5% ropivacaine group had shorter time of micturition exceeding 100 ml and lower voiding International Prostate Symptom Score than the 15 mg 0.5% ropivacaine group (*P *< 0.01).

**Conclusions:**

Single spinal anesthesia with 10 mg 0.5% ropivacaine not only provides satisfactory anesthetic effect for hemorrhoidectomy but also has less influence on postoperative uroschesis and is worthy of clinical application.

**Trial registration:**

The study was registered in the Chinese Clinical Trial Registry (http://www.chictr.org.cn; identifier: ChiCTR2,100,043,686) on February 27, 2021.

## Introduction

Anorectal diseases are common and include internal, external, and mixed hemorrhoids (1). Treatment for hemorrhoids patient is usually determined by preoperative clinical evaluation, and surgery is necessary for most symptomatic Grade III and IV patients (2, 3). Surgical procedures performed below the dentate line include simple hemorrhoidectomy, circumferential hemorrhoidectomy, and removal of thrombotic external hemorrhoids. The main operations at sites above the dentate line include procedures for prolapse and hemorrhoids (PPH) (4, 5). Although hemorrhoid surgery is a brief operation, anesthesia, anesthetic drugs, drug concentrations, and anesthesia level control are closely related to postoperative uroschesis (6, 7). Early postoperative activities, early voluntary urination can help patients recover quickly after surgery. The impulse of bladder filling and dilation is transmitted to the sacral spinal primary urination center, and then uploaded to the higher center of the cerebral cortex to produce the intention of urination (8). For hemorrhoid surgery, a single spinal block with ropivacaine, which is commonly used, blocks the S2-S4 parasympathetic nervous system that restraining the micturition reflex, causing postoperative urinary retention, and affecting the rapid postoperative recovery of patients (9, 10). This study was designed to investigate the effects of two doses ropivacaine that produce satisfactory analgesia, muscle relaxation and inhibit adverse reflexes on urinary retention in patients undergoing hemorrhoidectomy.

## Methods

### Patients and inclusion criteria

230 male patients, aged 20–60 years, who underwent anorectal surgery under ASA grade I–II, with single spinal anesthesia in our hospital between March 2021 and March 2022 were recruited. There were no significant preoperative physical or laboratory abnormalities. In order to maintain consistency, all patients included in this study will be treated with the PPH in the operating room, and the surgery was completed by the same group of surgeons. The dose of ropivacaine is invisible to patients and surgeons.

### Patients and exclusion criteria

Patients with serious cardiac or cerebrovascular disease and/or liver, kidney, and lung failure, spine diseases, benign prostatic hyperplasia (BPH), urethral stricture, urethral stones, neurogenic dysuria and abnormal bladder urination, abnormal lower limb movements, contraindications to intraspinal anesthesia, abnormal changes in the anesthesia method were excluded.

### Setting

**Figure 1:**
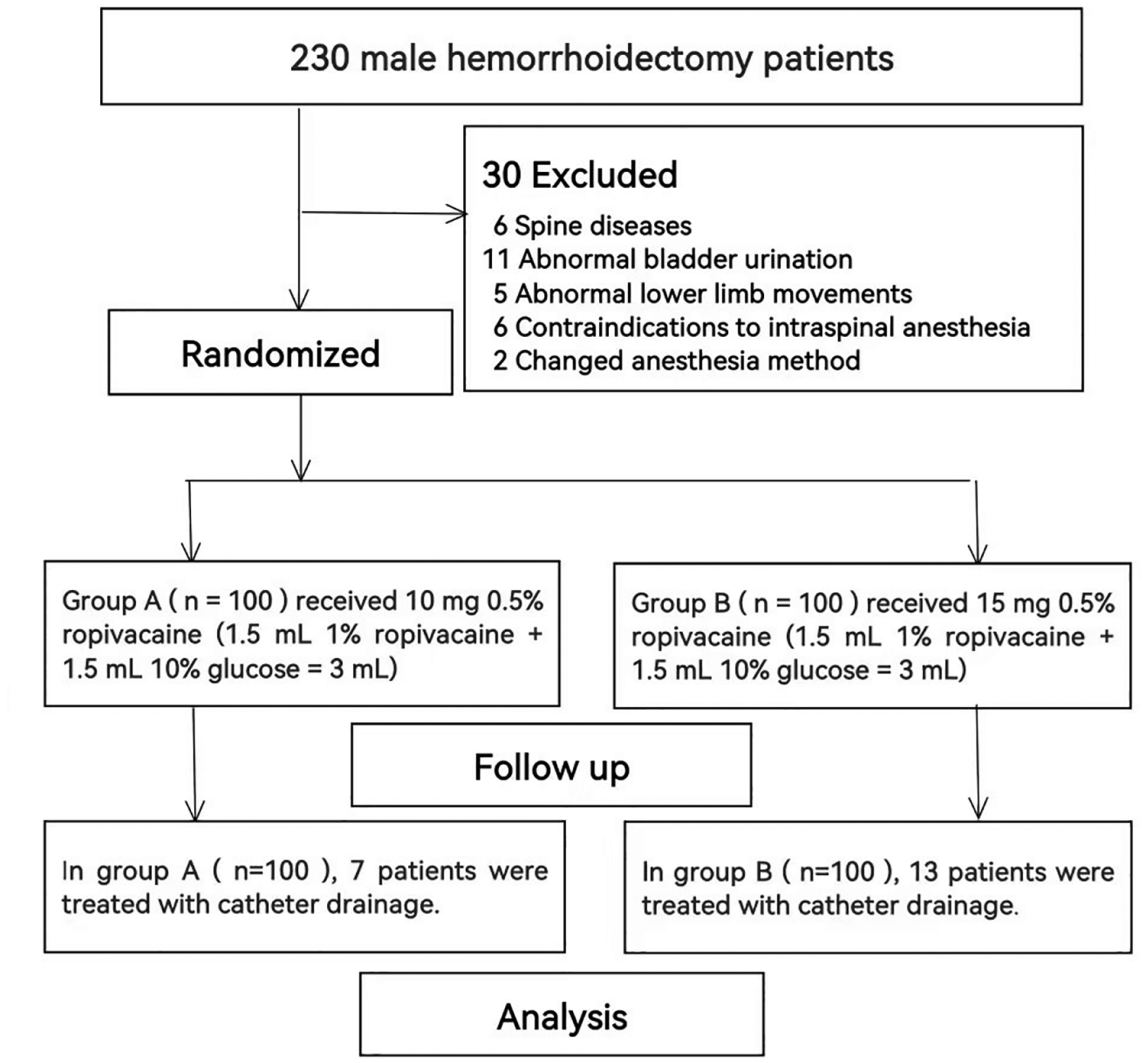
Flow of the participants in the study.

Finally, 200 cases of hemorrhoidectomy were obtained. Patients were randomly assigned to 2 groups using a random number table: Group A (*n* = 100) received 10 mg of 0.5% ropivacaine (1.5 ml 1% ropivacaine + 1.5 ml 10% glucose = 3 ml), and Group B (*n* = 100) received 15 mg of 0.5% ropivacaine (1.5 ml 1% ropivacaine + 1.5 ml 10% glucose = 3 ml) (Figure 1).

### Anesthesia management

The patients were forbidden to drink at 2 h and fasted at 6 h before surgery and were asked to empty their bladder before entering the operating room. Before the surgery, ECG, non-invasive blood pressure, and pulse oxygenation were monitored. At 20 min before anesthesia administration, 8 ml/kg of Ringer's lactate solution was infused, and 30 min later, 8 ml/kg/h solution was administered and maintained until the end of the operation. Patients in Group A were situated in the left side position, the area was disinfected, and 10 mg of 0.5% ropivacaine was injected upward after cerebrospinal fluid reflux for 30 s. After the injection, the patients were immediately returned to the supine position and then changed to the lithotomy position after 10 min of anesthesia. The patients' vital signs, pain perception, and motor block were then assessed. Patients in Group B were treated with 15 mg of 0.5% ropivacaine, and all other conditions were identical to those in Group A. A lithotomy position was used for the operation. At 6 h after the operation, the patients were given 2 sustained-release tablets of a compound preparation of ibuprofen and codeine (Qd).

### Observation indices

The observation indices included the ropivacaine dose, Numerical Rating Scale (NRS) score for anal pain, time required for lower limb muscle strength recovery (hours), anesthesia satisfaction at 48 h after the operation, time after the operation of voiding more than 100 ml (hours), usage of a postoperative indwelling catheter, and postoperative International Prostate Symptom Score (I-PSS) (11).

Pain degree was assessed using the anal pain NRS, which includes 11 ratings ranging from 0 to 10, with a total of 10 points. A higher anal pain score indicated more severe pain. The pain index scores were as follows: 0 indicated no pain; 1, 2, and 3 indicated mild pain; 4, 5, and 6 indicated moderate pain; 7, 8, and 9 indicated severe pain; and 10 indicated extreme pain.

Motor block was assessed using the modified Bromage score (12), and muscle strength grades were expressed as 0, I, II, III, and IV. Grade 0 indicated that patients could not bend their ankles, knees, or hips and could not lift their legs off the bed; Grade I indicated pliability of the ankles; Grade II indicated pliability of the ankles and knees; Grade III indicated pliability of the ankles, knees, and hips; Grade IV indicated pliability of the ankles, knees, and hips and patients could lift their legs off the bed.

The I-PSS (13) is the leading international standard for assessing the severity of symptoms in patients with BPH. The I-PSS is also a subjective indicator of the severity of lower urinary tract symptoms in BPH patients. Single spinal anesthesia using two doses ropivacaine blocked the S2-S4 parasympathetic nervous system, causing symptoms similar to BPH. The I-PSS was recorded within 2 days after hemorrhoidectomy. The I-PSS classification is as follows: total score 0–35; mild uroschesis 0–7; moderate uroschesis 8–19; severe uroschesis 20–35.

### Statistical analysis

According to Kreutziger (14), the incidence of urinary bladder catheterization was 6.3% in male patients. In our preliminary study, the incidence of urinary bladder catheterization was 12.0%. A sample size of 173 patients was calculated to have a power of 0.8 and a signiﬁcance level of 0.05 to detect a difference with PASS software (version 8.03; NCSS LLC, Kaysville, UT, United States). In consideration of a potential dropout, 230 male patients were recruited for the study. The data were analyzed using SPSS (version 25.0). Qualitative data are summarized as percentages, and quantitative data are expressed as means and standard deviations. T-test was performed to assess qualitative data, and a Chi-square test was used to examine differences in proportions. The Mann–Whitney *U*-test was used to analyze non-normal variables. With the statistical analysis, a value of *P* < 0.05 was considered statistically significant.

## Results

### General information

The general information of 200 male patients is listed in Table 1. The age, height, weight, operation time, anesthesia time and ASA grade were not significantly different between the two groups (*P *> 0.05, Table 1).

**Table 1:** Comparison of general information.

Variable	A group (*n* = 100)	B group (*n* = 100)	Statistical Test	*P*-value
Age (years)	43 ± 10	42 ± 11	*t* = 0.976	0.330
Height (cm)	165.9 ± 5.2	166.1 ± 5.0	*t* = 0.345	0.730
Weight (kg)	73.2 ± 8.8	71.0 ± 9.2	*t* = 1.782	0.076
Operation time (min)	20.62 ± 3.018	20.64 ± 3.099	*t* = 0.046	0.963
Anesthesia time (min)	30.87 ± 2.905	30.69 ± 2.837	*t* = 0.441	0.660
ASA (grade I/grade II)	25(25)/75 (75)	23 (23)/ (77)	*χ*^2^ = 0.110	0.741

### Anorectal pain block

Pain degree was assessed using the anal NRS, and satisfactory pain block was achieved during and after the operation. There was no significant difference in NRS scores among patients who received two doses ropivacaine for the operation (*P *> 0.05, Table 2). Patients with anal NRS (4–6) score were injected Tramadol 100 mg at 48 h (T10) postoperatively (Table 3), after which these patients got a painless state. A single hemorrhoidectomy was performed with 0.5% ropivacaine (10 mg or 15 mg), the anal sphincter exhibited good relaxation, and there was no obvious traction pain.

**Table 2:** Comparison of Numerical rating scale anal pain scores.

Time points	A group (*n* = 100)	B group (*n* = 100)	*t*	*P*–value
T1	5.0 ± 0.5	5.0 ± 0.4	0.520	0.604
T2	1.9 ± 0.5	1.9 ± 0.5	0.145	0.885
T3	0.0 ± 0.0	0.0 ± 0.0	/	/
T4	0.0 ± 0.0	0.0 ± 0.0	/	/
T5	0.0 ± 0.0	0.0 ± 0.0	/	/
T6	1.1 ± 0.3	1.1 ± 0.3	0.758	0.449
T7	2.0 ± 0.4	1.9 ± 0.4	1.559	0.121
T8	2.9 ± 0.3	2.9 ± 0.3	0.000	1.000
T9	3.8 ± 0.5	3.8 ± 0.5	1.318	0.189
T10	5.0 ± 0.6	4.9 ± 0.5	1.027	0.306

T1, time of entry; T2, spinal anesthesia time; T3, supine position 10 min after anesthesia; T4, operation start time; T5, operation end time; T6, 3 h after the operation; T7, 6 h after the operation; T8, 9 h after the operation; T9, 24 h after the operation; T10, 48 h after the operation.

**Table 3:** Comparison of lower limb muscle strength recovery time, urination after anesthesia and satisfaction at 48 h after anesthesia.

Variable	A group (*n* = 100)	B group (*n* = 100)	Statistical Test	*P*-value
Time to grade IV (hours)	3.13 ± 0.55	3.22 ± 0.51	*t* = 1.131	0.260
Number of patients tramadol injected	82 (82)	83 (83)	χ^2^ = 0.35	0.852
Urination			χ^2^ = 2.000	1.157
Urinate in physiological position	93 (93)	87 (87)	* *
Indwelling catheter drainage	7 (7)	13 (13)	* *
Satisfaction			*z* = 0.438	0.661
Very satisfied	8 (8)	6 (6)		
Relatively satisfied	28(28)	28 (28)		
Not very satisfied	60 (60)	61 (61)		
Not satisfied	4 (4)	5 (5)		

Time to Grade IV, pliability of the ankles, knees, and hips and patients could lift their legs off the bed in the number of hours.

### Urination after anesthesia

Pertinent intervention measures, cognitive education, and psychological counseling should be performed before surgery to correct misconceptions and improve the attitudes and compliance of patients. Creating a suitable urination environment and guiding the physiological position during urination after surgery can promote smooth urination in patients (15, 16). The ropivacaine dose was not associated with a statistically significant difference in postoperative urination rates between the 200 patients who used a physiological position or indwelling catheter drainage (*P *> 0.05), as shown in Table 3.

### Satisfaction with two doses ropivacaine at 48 h after spinal anesthesia

Single spinal anesthesia with 0.5% ropivacaine (10 mg or 15 mg) not only satisfied all patients during hemorrhoidectomy, produced satisfactory analgesia and muscle relaxation, and inhibited adverse reflexes, but also promoted early recovery of lower limb movement. There was no significant difference between the groups in the number of hours required for muscle strength recovery. And there was no significant difference in anesthesia satisfaction between the two groups (*P *> 0.05, Table 3).

### Time required for micturition exceeding 100 ml after single spinal anesthesia

This study included 200 patients, and 7 patients in Group A and 13 patients in Group B were treated with catheter drainage. The remaining patients urinated in a physiological position with the help of family members after their muscle strength had fully recovered. A daily urine volume of less than 100 ml is considered no urine in healthy people, and a statistical analysis of the time until urination of more than 100 ml (hours) in patients receiving two doses ropivacaine is shown in Table 4.

**Table 4:** Comparison of the time (h) required for urination of more than 100 ml after anesthesia and international prostate symptom scores.

Variable	A group (*n* = 93)	B group (*n* = 87)	T-value	*P*-value	95%CI
T11	5.82 ± 0.72	6.20 ± 0.62	3.773	<0.001	0.180–0.577
Total score	4.73 ± 1.19	11.78 ± 1.87	30.373	<0.001	6.592–7.509

T11, voiding more than 100 ml (h) after the operation; Total score, the I-PSS score within 2 days after hemorrhoidectomy.

### I-PSS 2 days after single hemorrhoidectomy

The I-PSS was recorded within 2 days after hemorrhoidectomy as shown in Table 4.

The two groups were injected with 1000 ml glucose and sodium chloride 6 h after the operation. The time required for urination of more than 100 ml was shorter in the 10 mg 0.5% ropivacaine group than that in the 15 mg 0.5% ropivacaine group (*P *< 0.001). And single spinal anesthesia with 0.5% ropivacaine (10 mg and 15 mg) provided rapid restoration of lower limb movement. The 10 mg 0.5% ropivacaine group had a lower total voiding I-PSS than the 15 mg 0.5% ropivacaine group Thus, the 10 mg 0.5% ropivacaine group had less urinary retention than the 15 mg 0.5% ropivacaine group (*P *< 0.001), as shown in Table 4.

## Discussion

The incidence rates of anal fissure, anal fistula, and hemorrhoids have increased with improvement of the standard of living. Additionally, the incidence of anorectal disease is increasing because of the preference for spicy and stimulating foods, increased work pressure, long sitting times, and irregular sleep times (17, 18). At present, surgery is often performed in the clinical setting. Generally speaking, epidural, sacral canal, and single spinal anesthesia are often used during surgery in the operating room, and single spinal anesthesia was better (19, 20). This study was performed to examine the effect of single spinal anesthesia on patients undergoing hemorrhoidectomy. The results showed that single spinal anesthesia in the spinous process space of L3-L4 will block the S2-S4 parasympathetic nervous system, cause postoperative urinary retention, and affect the postoperative recovery of patients. However, there are some risk factors of urinary retention after spinal anesthesia. Spinal anesthesia may influence urinary bladder functions, leading to urinary retention. In their analysis, Keita (21, 22) found that predictive factors for postoperative urinary retention were age ≥50 years, duration of surgery ≥60 min, duration of anesthesia ≥80 min, quantity of intraoperative fluids ≥750 ml, and bladder volume on entry in the postanesthesia care unit ≥270 ml. In the literature, Postoperative urinary retention was diagnosed when patients were unable to void and the volume of urine in the bladder exceeded 400–600 ml (23). However, the routine ultrasound examination of bladder volume was not carried out in our study. It was shown that detrusor contraction is abolished within a few minutes (2 to 5 min) after intrathecal administration of local anesthetic, and muscle contraction recovery depends on the duration of the sensory block above the 3rd and 4th sacral segments (24). Spinal anesthesia does not influence the functions of the bladder sphincter muscle. Common causes of urinary retention include paralysis of the bladder sphincter and insensitivity to the micturition reflex caused by single spinal anesthesia, reflex spasms of the urethral sphincter caused by incision pain after perineum surgery, changes in micturition posture after surgery, excessive fluid replacement, and mental and psychological factors (25, 26). Other studies have shown that factors that influence urinary retention after spinal anesthesia include the operation time and local anesthetic dosage (27–29). Therefore, this trial controlled other variables that affect urinary retention after single spinal anesthesia, and the effect of low-dose single spinal anesthesia on postoperative urinary retention was examined in patients with hemorrhoidectomy.

Local anesthetics block the production and conduction of all nerve impulses (peripheral or central, afferent or efferent, protuberant or cell body, terminal or synapse). The degree of block is related to the dose, concentration, nerve fiber type, and stimulation intensity of local anesthetics (30). Local anesthetics must be in direct contact with nerve tissue before they take effect. As the concentration increases, pain sensation disappears first, followed by cold and heat and then touch and deep sensation, and finally motor function disappears (31).

At present, ropivacaine is widely used for spinal canal anesthesia in clinical practice. The drug is an amide pure S-type l-hydrochloric acid isomer and has low central nervous system toxicity and cardiotoxicity. In moderate drug concentrations, ropivacaine can produce the separation of motor and sensory nerve block (32). Domestic studies have shown that a total dose of 10–22.5 mg of 0.5% ropivacaine is safe and reliable, and a lower dose should be selected for older patients (33). This anesthesia scheme has the advantages of satisfactory analgesia, stable intraoperative hemodynamics, and a low incidence of postoperative complications (34). The addition of glucose to low-dose ropivacaine can further improve the motor nerve block ability.

Finally, this study controlled other variables to examine the effect of single spinal anesthesia with 10 mg and 15 mg of 0.5% ropivacaine and 10% glucose on postoperative urinary retention in patients with hemorrhoidectomy. The results showed that the combination of 10 mg and 15 mg 0.5% ropivacaine and 10% glucose could meet the above three conditions for nerve conduction block. Additionally, this combination could meet the requirements for satisfactory analgesia and muscle relaxation and inhibition of adverse reflexes during hemorrhoidectomy without affecting the effect of lower limb movement on postoperative urinary retention. The micturition time of the 10 mg 0.5% ropivacaine group was shorter than that of the 15 mg 0.5% ropivacaine group (*P *< 0.01). The total I-PSS for micturition in the 10 mg 0.5% ropivacaine group was lower than that in the 15 mg 0.5% ropivacaine group (*P *< 0.01); that is, the I-PSS retention symptoms in the 10 mg 0.5% ropivacaine group were less severe than those in the 15 mg 0.5% ropivacaine group (*P *< 0.01).

## Limitations

Our study had several limitations. Firstly, we did not include ASA III patients, considering of the clinical practice efficacy and safety. Secondly, because of differences in the urinary system between male and female, this study only focused on male patients. Furthermore, our results only apply to patients undergoing procedure for PPH in the operating room. Previous studies have shown that local anesthesia haemorrhoids could also be treated in an outpatient setting safely and effectively without urinary retention (35). Whether the results of this study can be applied to female patients, outpatient setting and other operations, further investigation is warranted.

## Conclusions

Use of a single dose of 10 mg of 0.5% ropivacaine for spinal anesthesia not only provides satisfactory anesthetic effect for hemorrhoidectomy but is also associated with a short time until urination exceeding 100 ml when a physiological position is achieved after surgery and has little impact on urinary retention. Therefore, ropivacaine has good prospects for application.

## Data Availability

The raw data supporting the conclusions of this article will be made available by the authors, without undue reservation.
